# Deorphanization of novel biogenic amine-gated ion channels identifies a new serotonin receptor for learning

**DOI:** 10.1016/j.cub.2021.07.036

**Published:** 2021-10-11

**Authors:** Julia Morud, Iris Hardege, He Liu, Taihong Wu, Myung-Kyu Choi, Swaraj Basu, Yun Zhang, William R. Schafer

**Affiliations:** 1 MRC Laboratory of Molecular Biology, Francis Crick Avenue, Cambridge CB2 0QH, UK; 2 Department of Organismic and Evolutionary Biology, Harvard University, 52 Oxford Street, Cambridge, MA 02138, USA; 3 Centre for Brain Science, Harvard University, Cambridge, MA 02138, USA; 4 Department of Medical Biochemistry and Cell Biology, Institute of Biomedicine, Medicinaregatan 9A, University of Gothenburg, 405 30 Gothenburg, Sweden; 5 Department of Biology, KU Leuven, Naamsestraat 59, 3000 Leuven, Belgium

**Keywords:** C. elegans, ligand-gated ion channels, serotonin, dopamine, tyramine, learning, olfaction, behavioural plasticity

## Abstract

Pentameric ligand-gated ion channels (LGICs) play conserved, critical roles in both excitatory and inhibitory synaptic transmission and can be activated by diverse neurochemical ligands. We have performed a characterization of orphan channels from the nematode *C. elegans*, identifying five new monoamine-gated LGICs with diverse functional properties and expression postsynaptic to aminergic neurons. These include polymodal anion channels activated by both dopamine and tyramine, which may mediate inhibitory transmission by both molecules *in vivo*. Intriguingly, we also find that a novel serotonin-gated cation channel, LGC-50, is essential for aversive olfactory learning of pathogenic bacteria, a process known to depend on serotonergic neurotransmission. Remarkably, the redistribution of LGC-50 to neuronal processes is modulated by olfactory conditioning, and *lgc-50* point mutations that cause misregulation of receptor membrane expression interfere with olfactory learning. Thus, the intracellular trafficking and localization of these receptors at synapses may represent a molecular cornerstone of the learning mechanism.

## Highlights

• Characterization of five new monoamine-gated ion channels in *C. elegans*
• The serotonin-gated cation channel LGC-50 is required for aversive learning
• Pathogen exposure redistributes LGC-50 to synaptic processes
• Regulated trafficking of LGC-50 is critical for the learning mechanism

## Teaser

Ligand-gated ion channels mediate fast synaptic transmission, and their regulation is critical for behavioral plasticity. Morud et al. identify a serotonin-gated channel required for learned avoidance of pathogenic bacteria and implicate its regulated trafficking to synapses in the learning mechanism.

## Introduction

Synaptic plasticity, the selective strengthening or weakening of individual synaptic connections, is fundamental to the diverse forms of learning and memory in all animals. At the molecular and cellular levels, most forms of synaptic plasticity are thought to involve alterations in the abundance, density, or sensitivity of ionotropic neurotransmitter receptors at the postsynaptic membrane. These ionotropic receptors fall into two general types: the nicotinic acetylcholine receptors (nAChRs) from the Cys-loop family of pentameric ligand-gated ion channels (LGICs) and the tetrameric ionotropic glutamate receptors (GluRs). Although emphasis has been on the role of these receptors in synaptic plasticity mechanisms, increasing evidence suggests that the regulation of pentameric channels other than nAChRs is also important.^1^, ^2^, ^3^, ^4^ The highly conserved Cys-loop superfamily includes both excitatory and inhibitory channels gated by a large variety of ligands; in mammals, this group includes glycine, GABA receptors (GABA_A_Rs) and serotonin receptors (5HT_3_R), although in other animals, such as nematodes, this family also includes both inhibitory and excitatory channels for the same ligands.^5^ It is not well understood to what extent non-nAChR Cys-loop channels are important in the molecular mechanism that underpins learning and synaptic plasticity.

Due to the complexity of mammalian neural circuits, it is challenging to pinpoint the contribution of a single receptor to learning processes. One way to address this question is by using an anatomically simple and genetically tractable organism, such as the nematode, *C. elegans.* It has a small nervous system consisting of 302 neurons, whose connectivity has been completely mapped.^6^ Remarkably, the *C. elegans* genome contains over 100 different Cys-loop genes, more than double the number found in the human genome. These include channels homologous to nAChRs and GABA_A_Rs, as well as subfamilies that are unique to nematodes, including monoamine gated anion channels.^7^, ^8^, ^9^ For many of these nematode LGICs, their basic properties, including their activating endogenous ligands, is not known.

Despite this small nervous system, *C. elegans* is capable of performing both non-associative and associative learning.^3^^,^^10^ These aspects make it possible to study the role of individual channels and their contribution in different behaviors. For example, animals infected with pathogenic strains of bacteria, such as *Pseudomonas aeruginosa* strain PA14, learn to avoid their odorants, which are often attractive to naive animals.^3^^,^^11^ Neuronal ablation and genetic experiments indicate that learned avoidance of PA14 requires a neural pathway involving serotonergic chemosensory neurons called ADF.^3^^,^^11^ ADF synapses onto the interneurons AIZ, AIY, and RIA, all of which play critical roles in the neural circuit underlying olfactory response to bacterial odorants and learning.^4^^,^^11^^,^^12^ Previous work showed that the function of the serotonin-gated channel MOD-1, expressed in AIZ and/or AIY, regulates the aversive learning of PA14.^3^^,^^4^ However, it is not clear whether a serotonin signal is important for the role of RIA in learning and what receptor may mediate this function of neural plasticity.

Here, we have characterized and identified ligands for five previously orphan monoamine-gated nematode LGICs. These monoamine-gated LGICs are expressed in neurons postsynaptic to aminergic neurons, implicating them in fast aminergic neurotransmission. One of these, the serotonin-gated cation channel LGC-50, is required for serotonin-dependent pathogen avoidance learning and functions in interneurons critical for this process. We further show that, *in vivo*, the redistribution of LGC-50 receptors to neuronal processes is induced by pathogen exposure and the proper trafficking of the receptor is critical for learning.

## Results

### Deorphanization of novel amine-gated LGICs

**Figure 1:**
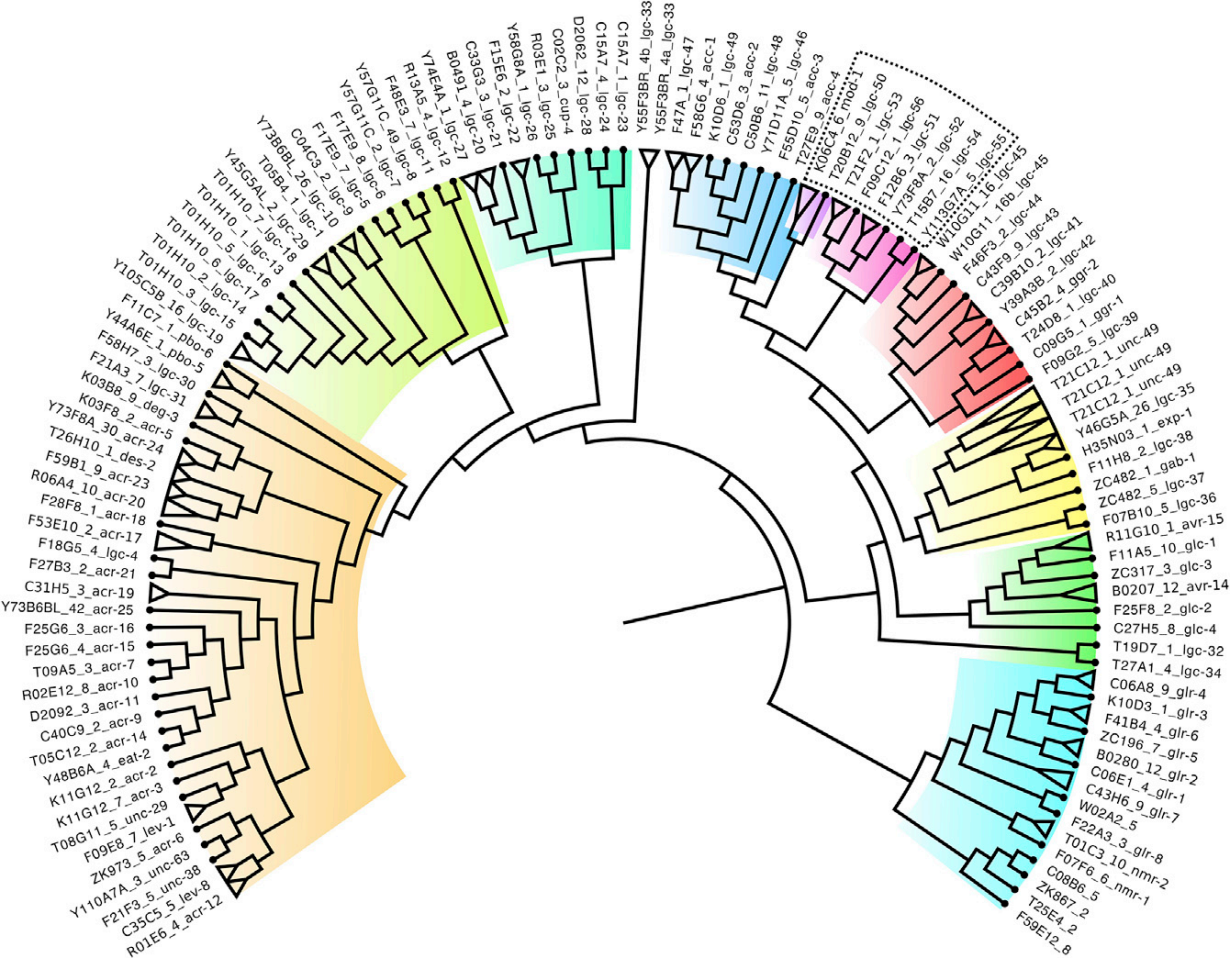
The superfamily of ligand-gated ion channel genes of *C. elegans* Cladogram of the LGIC genes of *C. elegans* colored by subfamily and the predicted amine-gated group highlighted in the dotted box. Orange, green, and turquoise, cationic nAChR-like; blue, anionic “ACC” acetylcholine gated; purple, serotonin gated; pink, amine gated; yellow, GABA gated; green, anionic glutamate gated; light blue, cationic glutamate gated. Isoforms were collapsed and shown as triangles. See also Table S1.

Although a number of monoamine receptors have been identified in *C. elegans*, many neurons receiving synaptic input from aminergic neurons express no known aminergic receptor.^13^ We reasoned that some of the uncharacterized Cys-loop LGICs might be receptors for monoamines. Three *C. elegans* LGCs have been previously described as monoamine gated,^7^^,^^8^^,^^14^ but many predicted LGICs had no characterized endogenous ligand. A phylogenetic analysis of 171 *C. elegans* LGIC genes (Table S1), based on the entire gene sequences, revealed the presence of a subfamily (Figure 1) that included the known monoamine-gated LGICs along with several uncharacterized channels. In line with previous studies, we found that *mod-1* and *lgc-50* diverge from other genes in this group.^5^

We aimed to deorphanize *C. elegans* LGICs without known ligands, focusing on 5 genes in the putative monoamine-gated group: *lgc-50*; *lgc-51*; *lgc-52*; *lgc-54*; and *lgc-56* (previously named *ggr-3*). Using heterologous overexpression in *Xenopus* oocytes and two-electrode voltage clamp recordings, we measured channel activity evoked by application of 11 monoamines and other neurotransmitters. The panel included all monoamines used in *C. elegans* (dopamine, serotonin, tyramine, and octopamine), classical neurotransmitters (acetylcholine, GABA, and glutamate), and other potential neuromodulators (betaine, tryptamine, histamine, and glycine). All ligands were screened at 100 μM, a concentration well above the expected half maximal effective concentration (EC_50_) value.

**Figure 2:**
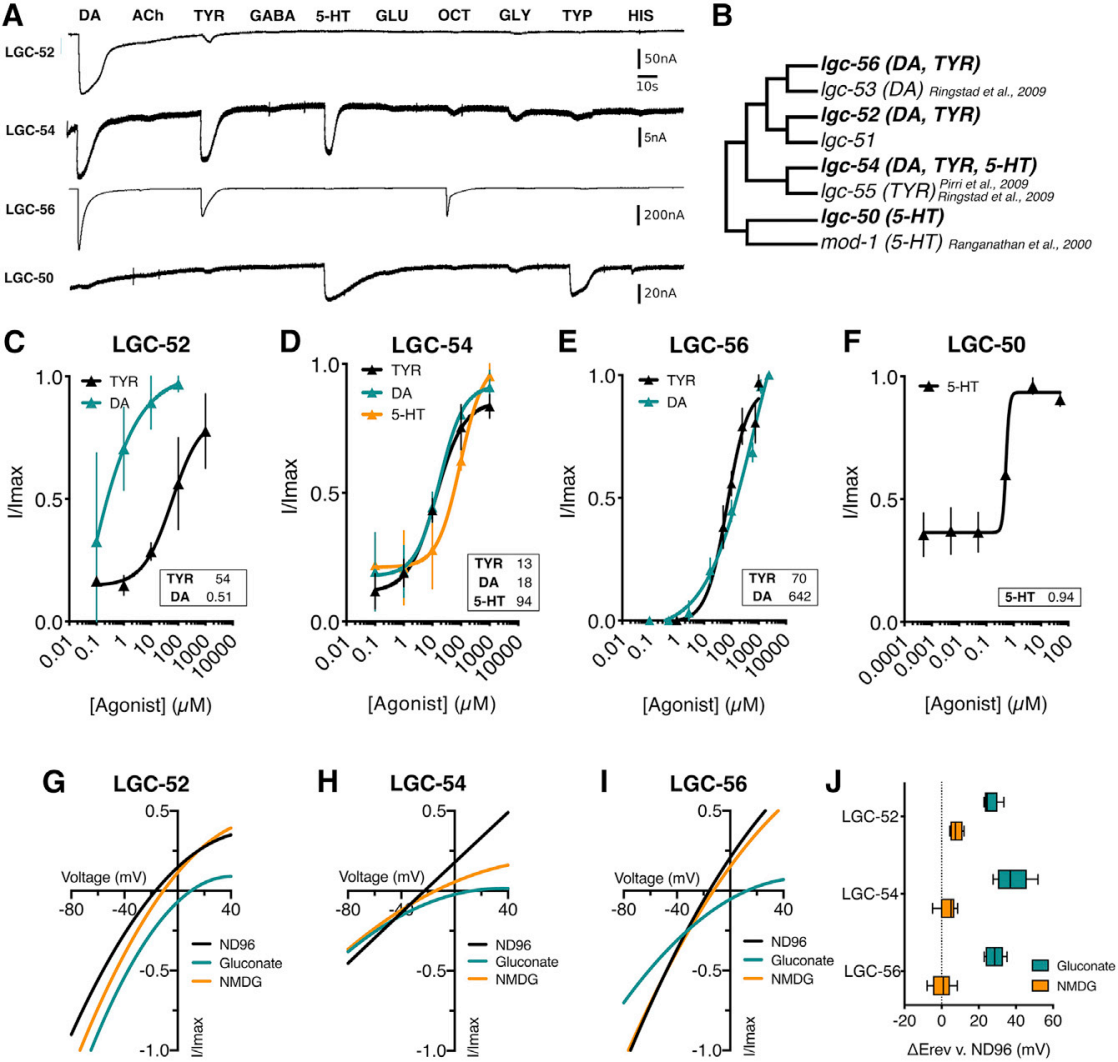
Deorphanization of ligand-gated ion channels (A) Representative traces of continuous recordings of *Xenopus* oocytes clamped at −60 mV expressing LGC-52, LGC-54, LGC-56, and LGC-50, exposed to 100 μM DA (dopamine), ACh (acetylcholine), TYR (tyramine), 5-HT (serotonin), GLU (glutamate), OCT (octopamine), GLY (glycine), TYP (tryptamine), and HIS (histamine). (B) Aminergic group based on phylogenetic analysis (Figure 1). (C–F) Agonist-evoked dose response curves from oocytes expressing aminergic LGICs. Curves fitted to Hill equation with variable slope using current normalized to I_max_ for each oocyte are shown. Insets show EC_50_ in μM. Error bars represent SEM for 3–9 oocytes. See Figure S1 for more representative traces. (G–I) Representative current-voltage relationships for oocytes expressing LGC-52, LGC-54, and LGC-56. The current was normalized to I_max_ for each oocyte and baseline current subtracted from agonist-evoked current, with agonist present at EC_50_ concentrations. (J) Average calculated from 4–10 oocytes for each construct of ΔE_rev_ in NMDG or gluconate versus in ND96. See also Figures S1 and S2.

In this initial screen, we identified ligands for four of the five LGICs. Three, LGC-52, LGC-54, and LGC-56, were activated by both dopamine and tyramine (Figures 2C–2E). LGC-54 also responded to 5-HT at high concentrations, shown by a high EC_50_ for 5-HT compared to dopamine and tyramine (Figure 2D). LGC-56 also displayed a small response to octopamine at very high (1 mM) concentrations (Figures 2A and S1A). LGC-52 showed a clear ligand preference for dopamine: its EC_50_ value was 100-fold lower for dopamine than tyramine, with dopamine also evoking larger peak currents. Although LGC-56 exhibited a higher sensitivity to tyramine over dopamine, shown by a significantly lower EC_50_ (insets Figures 2D and 2E), its peak responses to dopamine were larger. In contrast, LGC-50 was gated only by 5-HT, with an EC_50_ of 0.94 μM (Figure 2F), and the 5-HT metabolite tryptamine (Figure 2A). The final orphan channel of this subfamily, LGC-51, did not exhibit any specific currents in response to the ligands tested, although the protein appeared to be expressed in the oocytes due to failing viability 4 days after injection (Figure S1B).

We next investigated the ion selectivity of the newly deorphanized channels. We performed ion substitution experiments in oocytes expressing nematode LGICs and calculated the shift in reversal potential (Δ E_rev_) for a Na^+^ free (NMDG) or low Cl^−^ (Na gluconate) solution compared to a solution with high sodium and chloride (Figures 2G–2I). We found LGC-56, LGC-52, and LGC-54 to be anion-passing inhibitory channels, as oocytes expressing these receptors showed a significantly larger shift in low Cl^−^ solution than in Na^+^-free solution (Figure 2J). Anion selectivity of previously described LGICs depends on the conserved proline-alanine-arginine (PAR) motif in the M2-3 intracellular loop,^15^ which lines the channel pore and acts as a gate.^16^ All three of the newly deorphanized anion-selective receptors contain a PAR motif (Figure S2A).

### Aminergic LGCs are expressed postsynaptically to monoaminergic neurons

**Figure 3:**
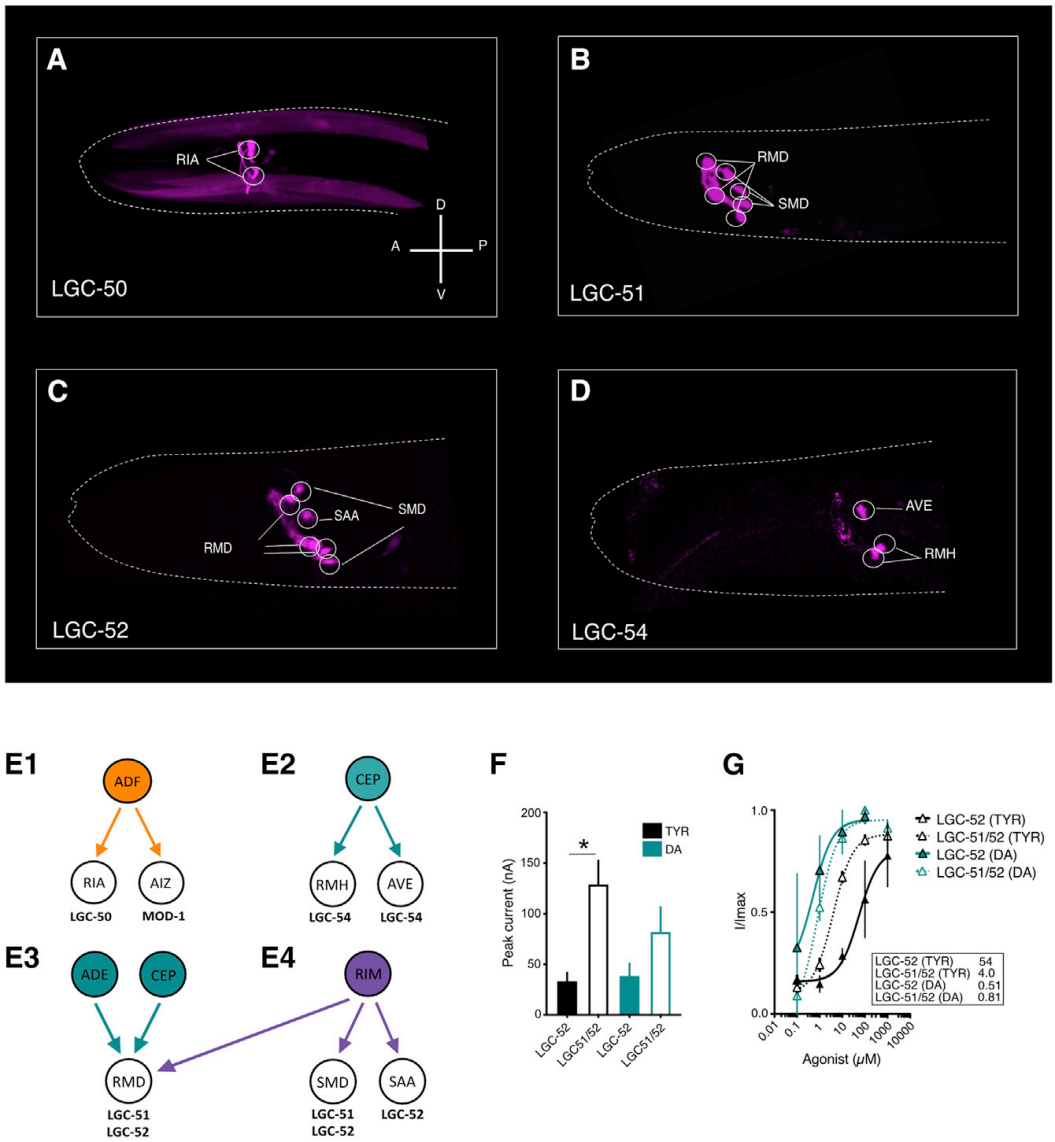
Novel amine-gated LGCs are expressed in major synaptic targets of aminergic neurons and identification of a heteromeric LGC (A–E) Reporter lines expressing intercistonically spliced mKate or GFP under gene-specific promotors. (A) Plgc-50::lgc-50::gDNA::SL2mKate. (B) Plgc-51::lgc-51::gDNA::SL2GFP. (C) Plgc-52::lgc-52::gDNA::SL2GFP. (D) Plgc-54::lgc-54::gDNA::SL2mKate. (E) (1–4) Graphical representation of neurons postsynaptic to aminergic neurons. (F) Oocyte peak current (nA) in response to application of 1 mM TYR or DA. Bars represent SEM of 5–14 repeats. (G) Agonist-evoked dose response curves from oocytes expressing LGC-51 or LGC-51/52. Curves fitted to the Hill equation with variable slope using current normalized to I_max_ for each oocyte are shown. Insets show EC_50_ in μM for DA and TYR. Error bars represent SEM for 4–9 oocytes. Scale bar indicates 100 μm. ^∗^p < 0.05 versus LGC-52 (TYR) by ordinary one-way ANOVA with Tukey’s multiple comparison correction. See also Figure S3.

Many of the principal synaptic targets of aminergic neurons have not been reported to express aminergic receptors.^13^ We speculated that these newly deorphanized aminergic channels might be expressed in some of these neurons and mediate aminergic synaptic transmission. To address this, we determined the expression pattern of each gene using fluorescent transcriptional reporters, containing both the upstream promoter region and the genomic DNA of each gene. We identified expression of the serotonin-gated channel *lgc-50* in the RIA interneurons (Figure 3A). RIA is a principal postsynaptic target of the serotonergic ADF neurons. The other major target of the ADFs are the AIZs, which have been shown to express the other serotonin-gated channel, *mod-1*.^17^ We did not observe any overlap in expression of *lgc-50* and *mod-1*, suggesting they have distinct roles in serotonergic communication. These results are consistent with serotonin-gated channels playing a key role in fast serotonergic neurotransmission.

We also observed expression of dopamine- and tyramine-gated channels in many neurons postsynaptic to dopaminergic and tyraminergic neurons. For example, *lgc-54* was expressed in two neuron pairs: AVE and RMH (Figure 3D). Both are major synaptic targets of the dopaminergic CEPs,^6^ and neither has been reported to express previously described dopamine receptors. *lgc-52* expression was observed in the RMD, SMD, and SAA neurons (Figure 3C), all major synaptic targets of the tyraminergic RIMs, with the RMDs also being targets of the dopaminergic CEPs and ADEs (Figure 3F). The expression for *lgc-56* was broader; we identified expression in BAG and ASH as well as a number of yet-unidentified neurons (Figure S3).

Interestingly, *lgc-51*, the only channel with no ligand response in our ligand screen, was expressed specifically in the RMDs and SMDs (Figure 3B), both of which also express its most closely related paralog *lgc-52*. We therefore hypothesized that LGC-51 and LGC-52 might form functional heteromers in these cells. To address this, we co-expressed LGC-51 and LGC-52 in *Xenopus* oocytes and compared these to oocytes expressing LGC-52 alone. We indeed observed currents in the LGC-51/52-expressing oocytes that were distinct (Figures 3F and 3G) from oocytes expressing LGC-52 alone. For example, although the EC_50_ for dopamine was similar between the homomer and the heteromer (0.51 μM and 0.81 μM, respectively), LGC-51/52 heteromers showed a much higher potency for tyramine (4 μM EC_50_) compared to the LGC-52 homomer (54 μM; Figure 3G). The tyramine-induced peak current was also significantly higher for the heteromer than LGC-52, although there was no significant effect on the dopamine peak current. Together, these results suggest that heteromerization of LGC-51 with LGC-52 predominately affects its gating efficiency by tyramine (Figure 3F), changing a channel with a strong preference for dopamine to one that is effectively activated by both tyramine and dopamine, at least *in vitro*.

### Diverse properties of dopamine- and tyramine-gated channels

**Figure 4:**
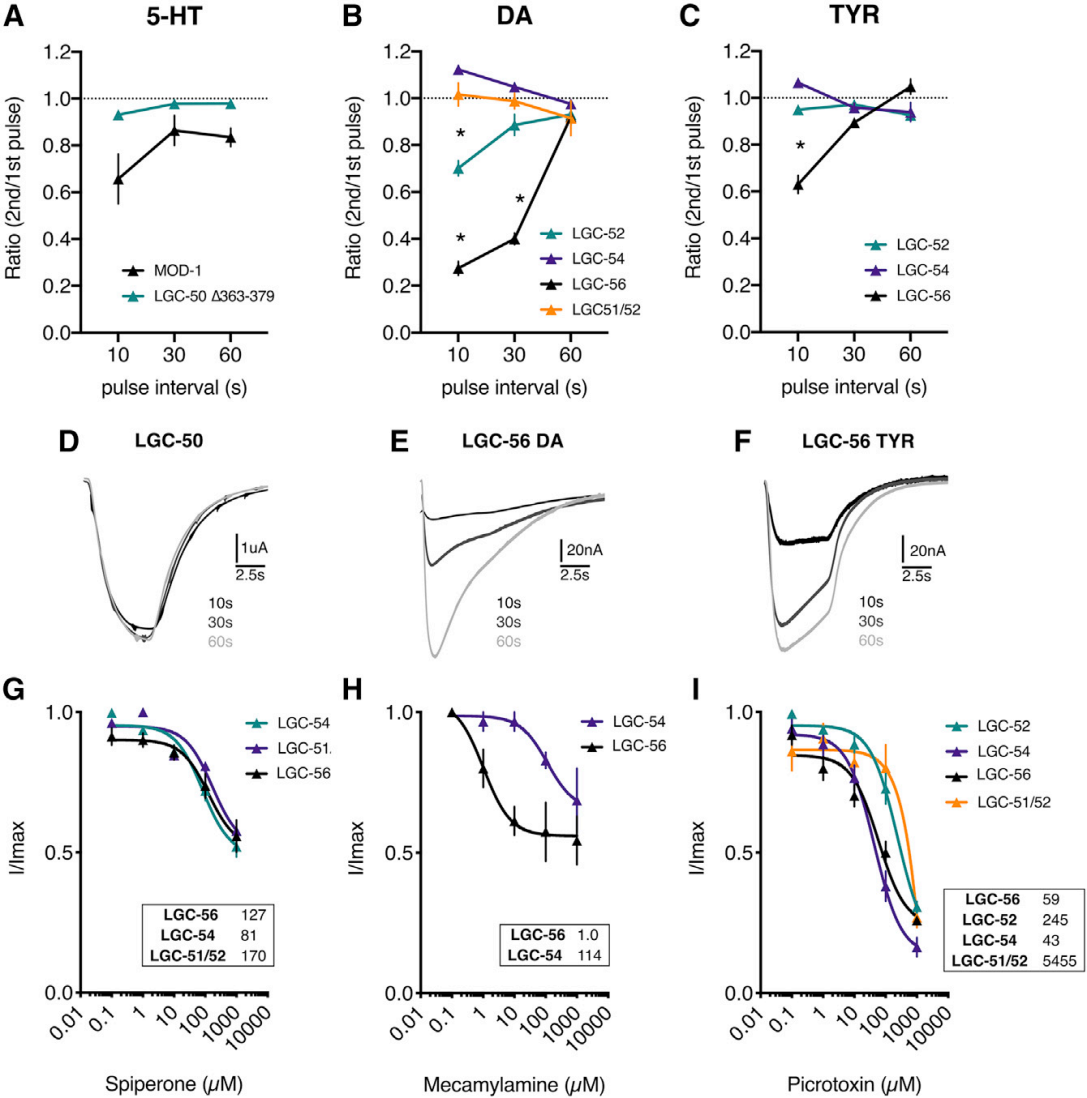
Differences in agonist occupancy and antagonistic profile for dopamine-gated channels (A) Oocyte peak current ratio of two 10-s agonist pulses with varying pulse intervals (s) during which the oocyte is washed with ND96 buffer. Agonist at the EC_50_ concentration for each channel is shown. Dashed line at ratio = 1. Error bars represent SEM of 3–8 repeats. ^∗^p < 0.05 compared to all other constructs at the interval, calculated by two-way ANOVA with Tukey’s multiple comparison correction. (B) Antagonistic inhibitory dose response curves from oocytes expressing aminergic LGICs. Agonist concentration remained constant at the respective EC_50_ for each channel. Curves fitted to the Hill equation with three-parameter slope using current normalized to I_max_ for each oocyte are shown. Insets show IC_50_ in μM. Error bars represent SEM for 4–9 oocytes.

We sought to dissect the properties of these dopamine- and tyramine-gated LGIC channels. First, we recorded the recovery time of the channels by exposing oocytes to multiple pulses of each agonist with varying ND96 wash intervals (Figure 4). For the serotonin-gated LGC-50 and MOD-1, there was no significant difference in recovery time between the channels (Figure 4A). In contrast, in response to multiple applications of dopamine, LGC-56 showed a significantly reduced peak current ratio at 10-s and 30-s intervals compared to LGC-54, LGC-52, and LGC-51/52 and did not recover to the maximal peak size until 60 s after the initial pulse (Figures 4B and 4E). At the 10-s pulse interval, LGC-52 also showed significantly slower recovery to initial pulse than LGC-54 and LGC-51/52 heteromer (Figure 4B). LGC-54 and the LGC-51/52 heteromer showed no significant reduction in peak ratios following repeated stimulation. When exposed to repeated tyramine application, only LGC-56 showed a significantly decreased peak ratio at 10-s pulse interval as compared to the initial pulse (Figures 4E and 4F).

We also investigated the antagonist profiles of three potential antagonists: mecamylamine; spiperone; and picrotoxin. These antagonists target different binding sites and have different specificity for vertebrate receptors. We found that mecamylamine, a nAChR blocker, thought to act by binding in the ligand binding region, partially blocked LGC-56 with a half-maximal inhibitory concentration (IC_50_) of 1 μM, whereas there was only a partial block of LGC-54 with an IC_50_ of over 100 μM (Figure 4H). This vast difference in IC_50_ suggests that the ligand binding domains of LGC-56 and LGC-54 may be structurally different. Spiperone, which preferentially binds dopaminergic receptors in mammals,^18^ led to a partial block of LGC-56, LGC-51/52, and LGC-54 with comparable IC_50_ values of 127 μM, 170 μM, and 81 μM, respectively (Figure 4G). Finally, picrotoxin, a well-characterized anion pore blocker,^19^ led to a large inhibition of currents, in particular for LGC-56 and LGC-54 with IC_50_ values of 59 μM and 43 μM, respectively. Interestingly, the IC_50_ of picrotoxin of the LGC-51/52 heteromer was an order of magnitude larger than that of the LGC-52 homomer (Figure 4I). This suggests that the pore structure and size of the LGC-52/51 heteromeric channel differs significantly from the LGC-52 homomer.

### LGC-50 is a cationic channel whose trafficking is regulated by its large intracellular domain

The newly deorphanized serotonin receptor LGC-50 was difficult to characterize due to small currents (on average 15 nA; Figure 2A). These currents were nonetheless dose dependent (Figure 2F) and absent in control oocytes (Figure S5A) and LGC-50 protein could be detected (Figure S4); in contrast, oocytes expressing another *C. elegans* ionotropic serotonin receptor MOD-1^14^ displayed much larger currents, indicating a higher degree of protein expression (Figure S5B). The intracellular loop between transmembrane helices 3 and 4 (M3/4) is widely accepted to be involved in the trafficking and proper cellular localization of LGICs.^20^^,^^21^ This domain is highly variable between LGICs and contains protein-protein binding sites and post-translational modifications.^22^ The two closely related serotonin-gated channels, *mod-1* and *lgc-50*, have 47% sequence identity outside of the M3/4 loop, compared to just 15% in the M3/4 loop (Figure S2B). Thus, we wondered whether the small currents observed for *lgc-50* might be a result of poor membrane localization of LGC-50 protein due to regulatory domains within the M3/4 loop.

**Figure 5:**
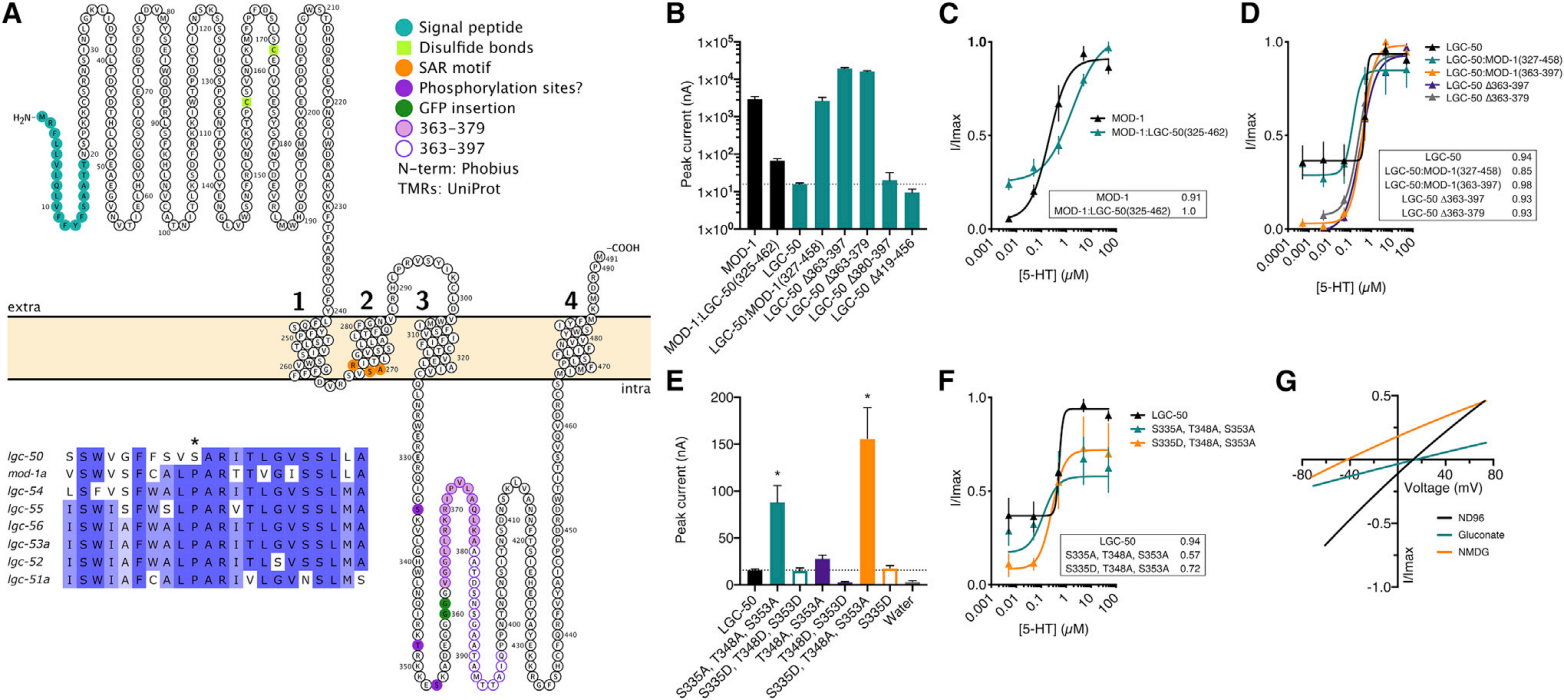
Identification of LGC-50 as a cationic channel and a binding motif for regulating surface localization (A) Topology diagram of LGC-50. Inset: alignment of “PAR” motif is shown. See Figure S2 for alignments. (B) Oocyte peak current (nA) in response to application of 5 μM 5-HT on different chimera version of MOD-1 and LGC-50. Bar represents mean + SEM of 2–26 repeats. Dashed line at wild-type LGC-50 peak current is shown. ^∗^p < 0.05 versus LGC-50; °p < 0.05 versus MOD-1 by ordinary one-way ANOVA with Dunnett multiple comparison correction. (C) 5-HT evoked dose response curves from oocytes expressing wild-type MOD-1 and MOD-1 mutants. See Figure S5 for representative traces of MOD-1 recordings. Error bars represent SEM of 8–12 oocytes. Curves fitted to the Hill equation with variable slope using current normalized to I_max_ for each oocyte are shown. Inset shows EC_50_ in μM. (D) 5-HT evoked dose response curves from oocytes expressing LGC-50 and MOD-1 mutants (LGC-50 wild type [WT] data have been replotted from Figure 2F). Error bars represent SEM of 3–6 oocytes. Curves fitted to the Hill equation with variable slope using current normalized to Imax for each oocyte are shown. Inset shows EC_50_ in μM. (E) Oocyte peak current (nA) in response to application of 5 μM 5-HT on phosphomimic versions of LGC-50. Bar represents mean + SEM of 2–30 repeats. Dashed line at WT LGC-50 peak current is shown. ^∗^p < 0.05 versus LGC-50, by ordinary one-way ANOVA with Dunnett multiple comparison correction. (F) 5-HT evoked dose response curves from oocytes expressing LGC-50 phosphomimic mutants (LGC-50 WT data have been replotted from Figure 2F). Error bars represent SEM of 3–6 oocytes. Curves fitted to the Hill equation with variable slope using current normalized to Imax for each oocyte are shown. Inset shows EC_50_ in μM. (G) Representative current-voltage relationships for oocytes expressing LGC-50 Δ363–379. Current was normalized to I_max_ for each oocyte and baseline current subtracted from agonist evoked current, with agonist present at EC_50_ concentration. See also Figures S4 and S5 and Table S2.

To investigate, we exchanged the intracellular M3/4 loop of LGC-50 with the equivalent region of MOD-1 (LGC-50:MOD-1 327–458) and expressed the chimera in oocytes. This led to a significant 175-fold increase in peak current relative to the native LGC-50; the peak current amplitude (2.6 μA) did not differ significantly to the peak current of wild-type MOD-1 (2.9 μA; Figure 5B). In the converse experiment, we exchanged the MOD-1 M3/4 loop for that of LGC-50 (MOD-1::LGC-50 325–462); this resulted in a significant 44-fold decrease in peak current to 66 nA, which in turn did not differ significantly to the peak current observed in wild-type LGC-50 (Figures 2B and S5B). The EC_50_ of the chimeric channels matched that of the recipient channel (Figures 5C and 5D). This suggests that the change in peak current conferred by the M3/4 region was due to altered membrane surface localization rather than changes to ligand binding efficacy or gating. Together, these data suggest that the M3/4 loop of LGC-50 might contain domains that are able to restrict plasma membrane trafficking.

To identify such domains, we tested the effects of deletion mutations in the LGC-50 M3/4 loop (Figure 5A). Deletions in the latter half of the loop, from 380 to 456, had no significant effect on the peak current compared to wild-type LGC-50 (Figure 5B). However, two deletions—Δ363–397 and the smaller deletion of Δ363–379—resulted in significant increases in peak current to 19 μA and 15 μA, respectively (Figure 5B). Indeed, the currents of these LGC-50 deletion mutants significantly surpassed the peak current achieved by either the LGC-50:MOD-1 chimera or wild-type MOD-1. Again, these deletion mutations showed similar dose dependency to wild-type LGC-50 (Figure 5D) as well as similar protein expression as wild-type LGC-50 (Figure S4), supporting the notion that these mutations alter cell surface trafficking rather than other properties of the channel. These data strongly suggest the presence of a functional domain within 16 amino acids of the M3/4 loop that results in the severe restriction of cell surface trafficking.

In addition to this region, we also investigated three upstream predicted phosphorylation sites. Phosphorylation of the M3/4 loop has previously been implicated in the trafficking and cell surface recycling of GABA_A_ receptors.^23^^,^^24^ The first site in the M3/4 loop of LGC-50, S335, is predicted to be a cdc2 site, and T348 and S353 are predicted protein kinase C (PKC) or PKA sites (Table S2).^25^^,^^26^ We introduced both phosphorylation-dead alanine mutations and phosphomimic aspartate mutations into these sites to assess their effect on receptor trafficking. We observed significant 5-fold and 10-fold increases in peak current amplitude for two mutation combinations: S335A, T348A, S353A and S335D, T348A, S353A. Dose dependency was not affected by either of these mutations, again suggesting an effect of the number of receptors at the surface rather than other changes to channel properties (Figures 5E and 5F).

As we were now able to induce efficient cell surface trafficking of LGC-50, we sought to determine the ion selectivity of the channel using the M34 deletion mutants through sodium and chloride ion substitution experiments. Interestingly, we observed that LGC-50Δ363–379 is selective for cations, with an average reversal potential shift in Na^+^-free solution (*N*-methyl-D-glucamine, NMDG) of −43 mV (Figure 5G). Thus, despite its phylogenetic proximity to GABA_A_ receptors and other anion-selective LGICs, *lgc-50* encodes an excitatory, cation selective serotonin receptor. It is worth noting that, unlike all other members of the aminergic LGIC group, LGC-50 does not contain the PAR region, which typically confers anion selectivity (Figure 5A, alignment inset);^15^ instead, the proline residue thought to be important for ion selectivity through the pore is substituted by a serine residue.

**Figure 6:**
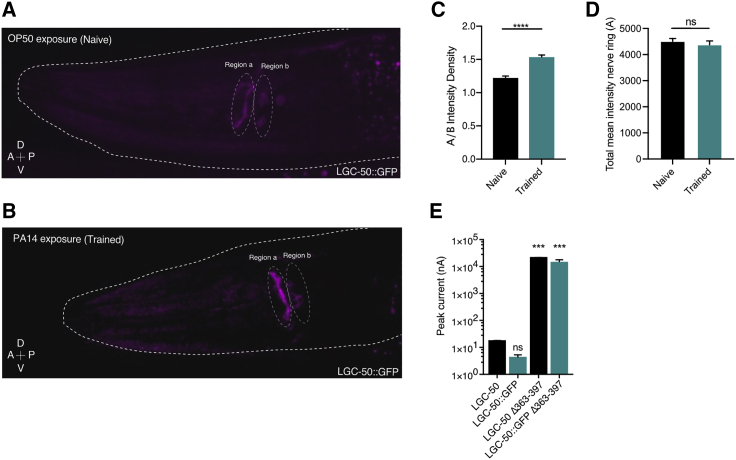
Exposure to pathogenic bacteria redistributes LGC-50 receptor protein (A and B) Representative image of an OP50 (A) and PA14 (B) exposed LGC-50::GFP(lj120)-tagged worms annotating the two regions a and b was used for intensity measurements. (C) Normalized fluorescent intensity for region a (nerve ring) as a ratio of region b (soma region). Bar represents mean + SEM; n = 48 OP50, 49 PA14. (D) Mean fluorescent intensity value of region a (nerve ring). (E) Oocyte peak current (nA) in response to application of 50 μM 5-HT in LGC-50 (untagged) or GFP-tagged LGC-50. Bar represents mean + SEM of 4–26 repeats. ^∗∗∗^p < 0.001; ^∗∗∗∗^p < 0.0001; ns, not significant. See Figure S4 for immunoblotting data of GFP-tagged LGC-50 wild-type versus LGC-50 Δ363–379.

To investigate the localization of LGC-50 *in vivo*, we generated CRISPR knockin worms expressing GFP-tagged LGC-50 protein from the *lgc-50* locus. Addition of GFP did not compromise the receptor’s ability to traffic to the plasma membrane, as both full-length and Δ363–379 GFP-tagged versions of the receptor generated currents of comparable size to the untagged receptor versions (Figure 6E). When we cultured GFP knockin animals on *E. coli*, we observed little expression of GFP-tagged LGC-50 in the nerve ring (Figure 6A), the site of most neuronal processes; instead, expression was mainly seen in cell bodies. However, when worms were exposed to the pathogenic bacteria *Pseudomonas aeruginosa* PA14, we observed a significant intensification of nerve ring expression of LGC-50::GFP compared to the fluorescence signal in the cell body region (Figures 6A–6D). Interestingly, no general increase in signal intensity was identified after PA14 exposure, which suggests that the treatment caused redistribution of already existing protein rather than increasing protein expression (Figure 6D). These results indicate that LGC-50 is redistributed from the cell body to neuronal processes in the nerve ring in response to pathogen exposure and infection, and the same mechanisms that limit LGC-50 trafficking in oocytes may also regulate receptor abundance in synaptic neuropil and participate in pathogen avoidance learning.

### LGC-50 in RIA is involved in aversive olfactory learning

**Figure 7:**
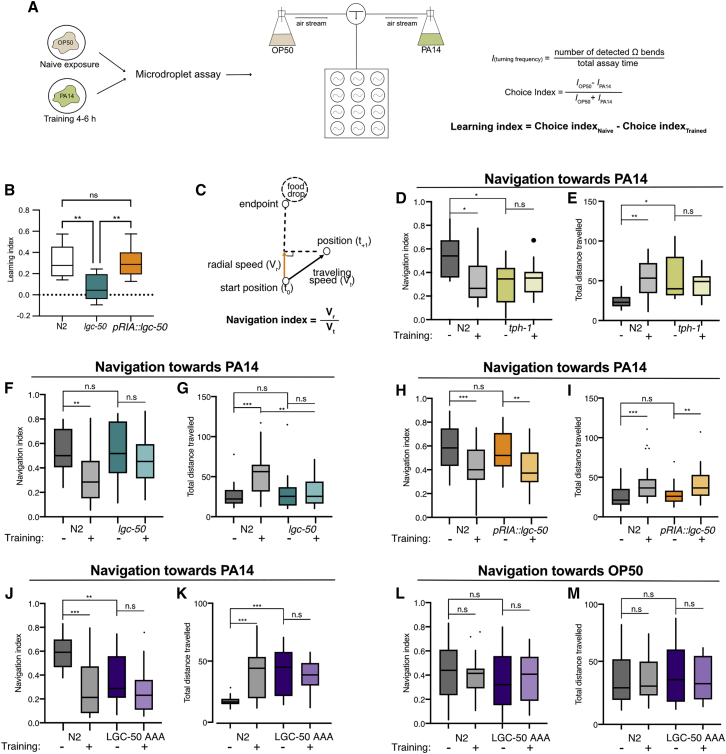
LGC-50 in RIA has a role in aversive olfactory learning (A) Schematic demonstrating the aversive olfactory training with pathogenic bacteria and the droplet assay testing learning. (B) N2 and lgc-50 mutants significantly differed in the droplet learning assay; this phenotype can be rescued under pglr-6 promotor (pRIA::lgc-50). lgc-50 mutant animals used were non-transgenic pRIA::lgc-50 offspring. n = 8 assays each genotype; one-way ANOVA with Tukey’s multiple comparisons test; ^∗∗^p < 0.01; ns, not significant. Data are shown in Tukey's boxplots.. (C) Schematic demonstrating navigation index in the chemotactic steering assay. (D–M) N2, *tph-1(mg280)*, l*gc-50(tm3712)*, RIA-specific rescue (*lgc-50(tm3712); pglr-6::lgc-50::SL2mKate2* referred to as *pRIA::lgc-50*), and triple phosphor-dead mutant (*lgc-50(lj155)* referred to as LGC-50AAA) animals in olfactory steering assay after exposure to either OP50 or PA14. Travel distance is measured in mm. Data are shown in Tukey’s boxplots, unless otherwise specified. ^∗^p < 0.05; ^∗∗^p < 0.01; ^∗∗∗^p < 0.001; n.s., not significant;by two-way ANOVA with Tukey’s multiple comparisons test. *tph-1*: n = 12 naive, n = 12 trained (N2 tested in parallel n = 12 naive and 12 trained); *lgc-50(tm3712)*: n = 23 naive, n = 24 trained (N2 tested in parallel n = 25 naive and 22 trained); *pRIA::lgc-50*: n = 35 naive, n = 40 trained (N2 tested in parallel n = 35 naive N2 and 33 trained N2); *lgc-50(lj155)* tested for PA14 odorants: n = 17 each group; *lgc-50(lj155)* for OP50 odorants: n = 16 naive, n = 15 trained (N2 tested in parallel n = 16 naive N2 and 15 trained N2). See also Figure S6.

Serotonin has been implicated in aversive learning of pathogenic bacteria by *C. elegans*. Naive *C. elegans* is attracted to odorants produced by pathogenic PA14; however, animals that have been exposed to and infected by the pathogen learn to reduce their preference for the odorants. The ADF-RIA connections represent a critical step in the learned aversion pathway defined by cell ablation experiments,^11^ and serotonin-deficient *tph-1* mutants are defective in learned pathogen avoidance (Figures 7D and 7E).^3^^,^^12^ As LGC-50 showed expression in the RIAs, we wondered whether LGC-50 might play a role in aversive olfactory learning in response to pathogenic bacteria.

To investigate this question, we examined the effect of an *lgc-50(tm3712)* deletion mutation on aversive learning. Using either two-choice assay^3^^,^^12^ or a droplet assay,^11^ which measure the olfactory preference of naive or trained worms for *P. aeruginosa* strain PA14 compared to *E. coli* OP50, we identified a robust learning defect in *lgc-50(tm3712)* animals compared to wild-type controls (Figures 7A, 7B, S6A, and S6B). We also used a previously established chemotaxis assay to measure the steering movement toward the odorants of PA14.^12^ The efficiency of olfactory chemotaxis was calculated using the navigation index (Figure 7C), as well as the total traveling distance (Figure 7; STAR Methods). Consistent with our previous findings, after training with PA14, wild-type worms reduced the navigation index in the chemotactic steering and traveled a significantly longer distance before reaching PA14, but the *tph-1(mg280)* mutant did not show a training-induced change in navigation index or traveling distance (Figures 7D and 7E). In contrast to wild-type animals, *lgc-50(tm3712)* null mutants showed no difference in navigation index or in traveling distance after training (Figures 7F and 7G). The *lgc-50(tm3712)* mutants expressing wild-type *lgc-50* selectively in RIA (Figure S6C) generated aversive learning similarly to wild type in chemotaxis assay, indicating that LGC-50 functions in the RIA neurons to promote aversive learning (Figures 7H and 7I). Expressing the wild-type *lgc-50* gene in RIA also rescued the learning defects of *lgc-50(tm3712)* mutants measured by the droplet assay (Figure 7B). The *lgc-50(tm3712)* animals displayed normal chemotaxis toward the odorants of *E. coli* OP50, the standard food source for *C. elegans*, under both naive and training conditions (Figure S6D), indicating intact chemotaxis ability. Furthermore, in a separate learning paradigm—thermotaxis^27^^,^^28^—we found no difference between wild type and *lgc-50(tm3712)* mutants (Figure S6A), demonstrating normal sensorimotor activity of *lgc-50(tm3712)* animals. Together, these results demonstrate that LGC-50 functions in RIA for aversive olfactory learning.

In principle, the phenotype of the *lgc-50*-null mutant could indicate a general or non-specific requirement for LGC-50-dependent neurotransmission for the acquisition of learned pathogen avoidance. Alternatively, because we had observed that pathogen exposure led to increased receptor expression in the nerve ring, we hypothesized that this regulated expression and/or membrane trafficking might be part of the learning mechanism itself. To investigate these possibilities, we evaluated worms carrying the three phospho-dead mutations (S335A, T348A, and S353A), *lgc-50(lj155)*, which based on oocyte experiments would be expected to have a high level of receptor expression in the nerve ring even in the absence of pathogen exposure. Indeed, we observed a strong olfactory learning defect in these animals, as training on PA14 had no effect on the navigation index or distance traveled in the chemotaxis to PA14 (Figures 7J and 7K). Meanwhile, these animals still displayed normal chemotaxis to non-pathogenic *E. coli*, before and after training (Figures 7L and 7M). Intriguingly, both trained and untrained *lgc-50(lj155)* animals resemble wild-type animals after training, suggesting that constitutive trafficking of LGC-50 receptors in the mutant mimics the trained condition in wild type. These results are consistent with the possibility that the plasticity mechanism underlying learned olfactory aversion involves the regulated localization of LGC-50 receptors to the membrane.

## Discussion

### Divergent properties of *C. elegans* monoaminergic LGICs

Here, we describe five new *C. elegans* LGCs activated by monoamines, including four anion channels activated by dopamine and tyramine and one cation channel activated by serotonin. These results reveal a remarkable evolutionary plasticity in the fundamental properties of LGICs. For example, LGC-54 responds to dopamine, tyramine, and serotonin in physiological ranges, yet its phylogenetically closest relative LGC-55 forms a tyramine-selective channel. Likewise, the closest paralog of LGC-56, a channel most potently activated by tyramine, is LGC-53, a dopamine-selective channel. Closely related channels also show divergent ion selectivity; the closest paralog of the serotonin-gated cation channel LGC-50 is MOD-1, a serotonin-gated anion channel. Thus, the *C. elegans* monoamine-gated LGIC subfamily shows remarkable diversification in the fundamental properties of ligand binding and ion selectivity. *C. elegans* as well as other invertebrates contain a number of uncharacterized LGIC families; it is interesting to speculate that these may also have evolved novel functional properties, possibly including novel activating ligands.

Even channels activated by the same monoamine show divergence in their expression pattern, kinetics, and ligand preference. For example, among the newly deorphanized dopamine- and tyramine-gated channels, dopamine activated LGC-52 at the lowest concentrations but elicited significantly larger peak currents for LGC-56 than LGC-52. Perhaps these receptors may be differently localized *in vivo*, with low-affinity LGC-56 receptors localized closer to release sites in the active zones of the synapses and higher affinity LGC-52 receptors possibly localized extrasynaptically, a phenomenon seen with glutamatergic AMPA receptors and nAChRs.^29^, ^30^, ^31^, ^32^ Receptors also differed in their response to repeated agonist stimulation and in their antagonist binding profiles, which may suggest structural differences in the ligand binding domains of these channels or differences in pore size between homomeric and heteromeric channels. In the future, the natural functional diversity of this ion channel family should provide a useful test bed to explore the relationship between LGIC structure and function.

### Regulated trafficking and localization of LGC-50 channels

Our analysis of serotonin-gated LGICs has provided mechanistic insight into the regulation of LGIC membrane trafficking and synaptic localization. The serotonin-gated cation channel, LGC-50, when heterologously expressed in *Xenopus* oocytes, shows limited membrane expression, in contrast to the closely related serotonin-gated anion channel MOD-1.^14^ Reciprocal domain swapping experiments demonstrated that this difference is specified by the cytoplasmic loop between the third and fourth transmembrane domains. Specifically, we found a domain of 16 amino acids in the intracellular M3/4 loop whose deletion significantly increased membrane trafficking without affecting dose dependency or ion selectivity. Interestingly, both human GABA_A_R β subunits and glycine receptors, which show significant homology to LGC-50 (25.84% with β1; 27% for GlyR) use similar molecular mechanisms to regulate cell surface localization and trafficking, with the M3/4 loop strongly implicated in regulating trafficking, assembly, and localization of the receptor.^33^^,^^34^

We also observed a potential role for phosphorylation in the fine control of LGC-50 plasma membrane expression. Preventing phosphorylation of two predicted PKC sites in the M3/4 loop of LGC-50 led to significant increases in serotonin-induced current without affecting dose dependency, whereas phosphomimic mutations led to a reduction. Again, this parallels previous work on GABA_A_ receptors,^23^^,^^35^, ^36^, ^37^ showing that PKC phosphorylation of sites within the M3/4 loop of GABA_A_R induces receptor internalization and affects synaptic plasticity at GABAergic synapses.^34^ Although interfering with phosphorylation had a modest effect on LGC-50 trafficking (estimated by peak currents) compared to the M3/4 deletion, we hypothesize that multiple mechanisms may regulate cell surface expression and localization; for example, the short deleted region may restrict surface expression, whereas phosphorylation may affect internalization as it does in related channels.^23^^,^^24^ Together, our results suggest significant mechanistic conservation in the regulation of LGIC trafficking across diverse phyla, which could be adapted to generate neural and behavioral plasticity.

### An excitatory serotonin-gated LGC plays a critical role in associative learning

We also identified a key role for LGC-50 in aversive learning and memory. Previous work demonstrated that learned avoidance of odorants given off by pathogenic bacteria following infection depends on serotonin and the RIA interneurons, which receive extensive serotonergic innervation.^6^^,^^11^ We find that *lgc-50*-null mutants are defective in pathogen avoidance learning, though their initial responses to pathogen odors are normal. This learning defect could be rescued by cell-specific expression of *lgc-50* in the RIA neurons, indicating that LGC-50 channels function in the RIA neurons to facilitate learned aversion. We also observed that exposure to pathogenic bacteria regulates the abundance of LGC-50 channels in synaptic neuropil; although little expression of LGC-50 was found in the nerve ring under normal growth conditions, its relative abundance in the nerve ring was enhanced following infection with pathogenic bacteria. Moreover, animals carrying phospho-dead mutations that increase plasma membrane localization *in vitro* were insensitive to aversive training, with unconditioned animals showing a similar olfactory preference to those conditioned by pathogen. Thus, we speculate that learning-induced redistribution of LGC-50 could play a role in memory formation.

How might LGC-50’s activity remodel the olfactory circuit to alter odorant preferences following pathogen training? Previous work has indicated that learned pathogen aversion depends on the serotonergic ADFs and their synaptic targets, the RIAs. Functional analyses suggest that, following training, RIA acts to inhibit the steering to pathogen odors through RIA synapses onto motorneurons.^11^^,^^12^ Our results suggest that induced expression of LGC-50 in the process of RIA, together with training-regulated serotonin signal,^3^^,^^38^ is an important mechanism for the mobilization of this aversive pathway following training. Previous work has shown that another serotonin-gated LGIC, MOD-1, acts in a different group of interneurons to regulate aversive olfactory learning.^3^^,^^4^ Together with LGC-50, these findings together highlight the critical role of serotonin-gated channels in neural plasticity. Interestingly, a recent study showed that insulin signaling, acting through the transcription factor DAF-16/FOXO, is also involved in learned avoidance of pathogenic bacteria in *C. elegans*.^39^^,^^40^ Comprehensive analysis of DAF-16 targets in the nervous system showed no evidence that LGC-50 is regulated by the insulin pathway.^41^ Thus, we suggest that both insulin and serotonin signaling regulate pathogen avoidance learning, but they likely act independently.

The results described here provide further insight into the roles of pentameric LGICs in learning and memory. Mammalian 5-HT_3_ receptors, which like LGC-50 are serotonin-gated cation channels, have been implicated in various forms of learning and behavioral plasticity. For example, 5-HT_3_ receptors have been shown to play a key role in reward pathways, with their insertion at synapses between the dorsal raphe nuclei and the ventral tegmental area, promoting dopamine release.^2^ Moreover, regulation of 5-HT_3_ receptor expression and abundance has been implicated in fear extinction.^1^ In addition, changes in the expression of 5-HT_3A_ receptors in the mouse visual cortex are important for cross-modal plasticity following sensory loss.^42^ The molecular mechanisms by which 5-HT_3_ receptor activity is regulated to generate synaptic plasticity in these examples are currently not well understood. In the future, it will be interesting to investigate whether regulated trafficking mechanisms similar to those in *C. elegans* may play a similar role in other organisms.

## STAR★Methods

### Key resources table

**Table:** 

REAGENT or RESOURCE	SOURCE	IDENTIFIER
**Antibodies**		

Rabbit polyclonal anti-GFP HRP conjugated	ThermoFisher	CAT #A10260, RRID:AB_2534022

**Bacterial and virus strains**		

*E. coli* OP50	Caenorhabditis Genetics Center	OP50
*Pseudomonas aeruginosa* PA14	Kim et al.^43^	Reference strain 14

**Chemicals, peptides, and recombinant proteins**		

5-HT	Tocris Bioscience	N/A
Dopamine	Sigma-Aldrich	N/A
Tyramine	Sigma-Aldrich	N/A
Octopamine	Sigma-Aldrich	N/A
Acetylcholine	Sigma-Aldrich	N/A
Glutamate	Sigma-Aldrich	N/A
Glycine	Sigma-Aldrich	N/A
GABA	Sigma-Aldrich	N/A
Histamine	Sigma-Aldrich	N/A
Tryptamine	Sigma-Aldrich	N/A
Picrotoxin	Tocris Bioscience	N/A
Spiperone	Tocris Bioscience	N/A
Mecamylamine	Tocris Bioscience	N/A

**Experimental models: organisms/strains**		

*C. elegans* var Bristol. N2	Caenorhabditis Genetics Center	N2
*lgc-50(lj154) III*	This study	AQ 4887
*lgc-50(lj155) III*	This study	AQ 4897
*lgc-50(lj157) III*	This study	AQ 4875
*lgc-50(lj120) III (CRISPR GFP insert pJML063)*	This study	AQ 4637
*ljEx1212 [plgc-56(3kb)::lgc-56(gDNA)::SL2 mKate2(pJML015); punc-122::GFP; pcDNA3.1]*	This study	AQ4314
*ljEx1218 [plgc-50(3kb)::lgc-50(gDNA)::SL2 mKate2(pJML025); punc-122::GFP; pcDNA3.1]*	This study	AQ4324
*lgc-50(tm3712) III outcrossed 8 generations*	This study	AQ4347
*him-5(e1490); IjEx1301[plgc-51::gDNA lgc-51 3*'*UTR::SL2 mKate2(pJML027);punc-122::GFP; pcDNA3.1]*	This study	AQ4531
*lgc-50(tm3712) III; IjEx1307 [pglr-6::lgc-50 (gDNA):: SL2 mKate2(pJML039); punc-122::GFP; pcDNA3.1]*	This study	AQ4552
*ljE*x*1328 [plgc-54::lgc-54(cDNA)::SL2 mKate2(JML051); punc-122::GFP; pcDNA3.1]*	This study	AQ4601
*ljEX1330 [plgc-52::lgc-52(cDNA)::SL2 mKate2(JML061); punc-122::GFP; pcDNA3.1]*	This study	AQ4612
*him-5(e1490); ljEx1340 [plgc-51::* *lgc-51(gDNA)::SL2 GFP(pJML064); punc-122::RFP]*	This study	AQ4638
*otIs669[NeuroPAL] V*	Hobert Lab^44^	OH15262
*lgc-50(lj155) III, bc3x (AAA phospho mutant)*	This study	AQ4897
*lgc-56::SL2 mNeonGreen(syb2794*) II	This study, by SUNYBiotech, Fuzhou, China	AQ4951
*tph-1(mg280)II*	Caenorhabditis Genetics Center	MT15434

**Recombinant DNA**		

Plasmid: KSM: *lgc-50 (cDNA)*	This study	pJML002
Plasmid: KSM: *lgc-50 (cDNA, S335D, T348D, S353D)*	This study	pIH129
Plasmid: KSM: *lgc-50 (cDNA, S335A, T348A, S353A)*	This study	pIH128
Plasmid: KSM: *lgc-50 (cDNA, S335D, T348A, S353A)*	This study	pIH134
Plasmid: KSM: *lgc-50 (cDNA, T348A, S353A)*	This study	pIH130
Plasmid: KSM: *lgc-50 (cDNA, T348D, S353D)*	This study	pIH131
Plasmid: KSM: *mod-1 (cDNA)*	This study	pIH124
Plasmid: KSM: *lgc-51a (cDNA)*	This study	pJML003
Plasmid: KSM: *lgc-52 (cDNA)*	This study	pJML004
Plasmid: KSM: *lgc-54 (cDNA)*	This study	pJML069
Plasmid: KSM: *lgc-56 (cDNA)*	This study	pJML007
Plasmid: KSM: *lgc-50 (cDNA, Δ363-379)*	This study	pIH142
Plasmid: KSM: *lgc-50 (cDNA, Δ380-397)*	This study	pIH143
Plasmid: KSM: *lgc-50 (cDNA, Δ419-456)*	This study	pIH144
Plasmid: KSM: *mod-1:lgc-50(325-462) (cDNA)*	This study	pIH145
Plasmid: KSM: *lgc-50:gfp (cDNA, Δ363 - 397, GFP in M3/4 loop)*	This study	pIH148
Plasmid: KSM: *lgc-50 (cDNA, S353D)*	This study	pIH141
Plasmid: KSM: *lgc-50:mod-1(327-458) (cDNA)*	This study	pIH125
Plasmid: KSM: *lgc-50 (cDNA, Δ363 - 397)*	This study	pJML138
Plasmid: KSM: *lgc-50:gfp (cDNA, GFP in M3/4 loop)*	This study	pJML091
Plasmid: pDEST: *plgc-51(2.5kb)::lgc-51(gDNA)::SL2 gfp*	This study	pJML064
Plasmid: pDEST: *plgc-52(2.5kb)::lgc-52(cDNA)::SL2 gfp*	This study	pJML065
Plasmid: pDEST: *plgc-52(2.5kb)::lgc-52(cDNA)::SL2 mKate2*	This study	pJML061
Plasmid: pDEST: *plgc-50(3kb)::lgc-50(gDNA)::SL2 mKate2*	This study	pJML025
Plasmid: *pDEST: plgc-51(2.5kb)::lgc-51(gDNA)::SL2 mKate2*	This study	pJML027
Plasmid: pDEST: *plgc-56(3kb)::lgc-56(gDNA)::SL2 mKate2*	This study	pJML015
Plasmid: pDEST: *plgc-54(3kb)::lgc-54(cDNA)::SL2 mKate2*	This study	pJML051
Plasmid: pDD380: *lgc-50 cDNA::gRNA in TM3-TM4 loop::3' and 5' homology arms::loxP (pDD363)::GFP (pDD372)::* *NT-tag donor (pMLS288)*	This study	pJML063
Plasmid: pDEST: *pglr-6(2.1kb)::* *lgc-50(gDNA):: SL2 mKate2*	This study	pJML039

**Oligonucleotides (see**Table S3**)**		

**Software and algorithms**		

Graphpad	GraphPad Software	Prism 8
Robocyte2+	Multichannel Systems	https://www.multichannelsystems.com/products/roboocyte2
Stimfit	Physiological Institute, University of Freiburg	http://www.stimfit.org/Home.html∼
WinWCP (Strathclyde Electrophysiology Software)	University of Strathclyde	http://spider.science.strath.ac.uk/sipbs/software_ses.htm
FigTree	Rambaut^45^	http://tree.bio.ed.ac.uk/software/figtree/
Affinity Designer	Serif Labs	https://affinity.serif.com/en-us/
Fiji / ImageJ	Schneider et al.^46^	https://imagej.nih.gov/ij/
RAxML v.8	Stamatakis^47^	https://academic.oup.com/bioinformatics/article/30/9/1312/238053
trimAI	Comparitive Genomics Group^48^	http://trimal.cgenomics.org
Python scripts can be found on GitHub at hiris25/TEVC-analysis-scripts	This paper	https://doi.org/10.5281/zenodo.5095181

**Other**		

Robocyte2	Multichannel Systems	https://www.multichannelsystems.com/products/roboocyte2
Roboinject	Multichannel Systems	https://www.multichannelsystems.com/products/roboinject

### Resource availability

#### Lead contact

Further information and requests for *C. elegans* strains and plasmids is to be sent to and will be fulfilled by the lead contact William R Schafer, wschafer@mrc-lmb.cam.ac.uk

#### Materials availability

Materials generated in this study, including strains, plasmids and clones, are freely-available from the lead contact upon request.

### Experimental model and subject details

#### *C. elegans*

Unless otherwise specified, worms were maintained at 20°C on nematode growth medium (NGM) plates seeded with bacterial *E. coli* (strain OP50). Transgenic lines were generated by injection of plasmid DNA into the gonad of day 1 adult hermaphrodites. Offspring with stable arrays were selected. Mutant strains generated by CRISPR were outcrossed at least three times, mutant strains obtained from million mutation project^49^ or by UV transgene integration were outcrossed at least six times, all to our laboratory stock of wild-type (N2). For a complete list of strains and transgenes used in this study see key resources table.

#### *Xenopus laevis* oocytes

Defolliculated *Xenopus laevis* oocytes were obtained from EcoCyte Bioscience (Dortmund, Germany) and maintained in ND96 (in mM: 96 NaCl, 1 MgCl_2_, 5 HEPES, 1.8 CaCl_2_, 2 KCl) solution at 16°C for 3-5 days.

### Method details

#### Molecular biology

*C. elegans* cDNA sequences were cloned from wild-type N2 worm cDNA generated by reverse transcription PCR using Q5 polymerase (NEB, MA, USA) from total worm RNA. Ion channel cDNA sequences for *Xenopus* oocyte expression were cloned into the KSM vector backbone containing *Xenopus* β-globin UTR regions and a T3 promoter. *C. elegans* gDNA sequences were cloned from wild-type N2 worm gDNA. Transgene expression was verified by GFP or mKate2 expression, either fused to the protein, driven on the same plasmid after an intercistronic splice site (SL2 site), or co-injected with *Punc-122*. Promoter sequences consisted of gDNA sequence approximately 2-3kb upstream of the start site of the gene. Subcloning was performed using HiFi assembly (NEB, MA, USA), IVA (*in-vivo* assembly, García-Nafría et al.^50^) or the Multisite Gateway Three-Fragment cloning system (Thermo Fisher Scientific, CA, USA) into pDESTR4R3II. Site-directed mutagenesis was performed using the KLD enzyme mix (NEB, MA, USA) or using IVA^50^. For full list of primers used, see Table S3.

#### CRISPR/CAS9-mediated gene manipulation

Genetic modifications including deletions and point mutations were made by following the Dokshin et al.^51^ protocol. gRNA and ssODN were ordered from Sigma (Merck group, Darmstadt, Germany), a list of sequences is provided in Key resources table. Endogenous tagging of *lgc-50* with GFP was carried out using the SapTrap protocol^52^^,^^53^ where GFP or mNeonGreen was added in the cytosolic M3/4 loop of the protein. The following allele were generated by SunyBiotech (Fuzhou, China) using CRISPR/Cas9-based genome editing: *lgc-56::SL2 mNeon Green(syb2794).* Sequences of plasmids and worm strains can be found in key resources table.

#### RNA synthesis and microinjection

5'-capped cRNA was synthesized *in vitro* using the T3 mMessage mMachine transcription kit according to manufacturer’s protocol (Thermo Fischer Scientific, CA, USA). RNA was then purified using the GeneJET RNA purification kit (Thermo Fischer Scientific, CA, USA) prior to cRNA injection. Defolliculated *Xenopus* oocytes were placed individually into 96 well plates and injected with 50nL of 500ng/μL RNA using the Roboinject system (Multi Channel Systems GmbH, Reutlingen, Germany). When two constructs were injected the total RNA concentration remained 500ng/μL, with a 1:1 ratio of the components. Injected oocytes were incubated at 16°C in ND96 until the day of recording, typically between 3-5 days post injection.

#### Two-Electrode Voltage Clamp (TEVC)

Two-electrode voltage clamp recordings were carried out using the Robocyte2 recording system or a manual TEVC set up, using an OC-725D amplifier (Multi Channel Systems, Reutlingen, Germany) and paired with a custom-made recording chamber and agar bridges from reference and bath electrodes. Glass electrodes were pulled on a P-1000 Micropipette Puller (Sutter, Ca, USA) with a resistance ranging from 0.7-2 MΩ, pipettes, containing AgCl wires, were backfilled with a 3 M KCl solution for manual recordings and 1.5M KCl and 1 M acetic acid for Robocyte2 recordings. Oocytes were clamped at −60mV unless stated otherwise. Continuous recordings were taken during application of agonists and antagonists at 500 Hz. Data was recorded using WinWCP or RoboCyte2 control software, manual data was filtered at 10 Hz.

Dose response curves were calculated from the peak current during a 10 s agonist stimulation in ND96 solution, with a 60 s ND96 wash in between doses. Data was gathered over at least two occasions, using different batches of oocytes. Normalized dose response data was fitted to a nonlinear curve using a four parameters variable slope and the EC_50_ and Hill slope was calculated. All further recordings were carried out with the agonist at its EC_50_ concentration unless stated otherwise. Ion selectivity was determined using a voltage ramp protocol of 20mV/s ranging from −80mV to +60mV in the presence or absence of the primary agonist in three different solutions: ND96, NMDG (Na^+^ free) and Na Gluconate (low Cl^-^) solutions. Normalized ramp curves were fitted to a linear regression line and the x intercept was compared between solutions to calculate an average E_rev_ from 4-5 oocytes. Antagonist dose response curves were calculated from the peak current during a 10 s agonist + antagonist stimulation in ND96 solution, with the agonist concentration remaining constant. Repeated agonist stimulus protocols were carried out by measuring the peak current during a 10 s agonist stimulation at three wash intervals, 10 s, 30 s and 60 s. Kinetic measurements were calculated from a 60 s agonist perfusion. Python scripts for TEVC analysis can be found at on GitHub at hiris25/TEVC-analysis-scripts and on Zenodo https://doi.org/10.5281/zenodo.5095181.

#### Confocal and Cell ID

Worms were mounted onto an 2% agarose pad and immobilised using 75 mM NaAzide in M9. Images were acquired using a Leica SP8, with a 63x objective, for further analysis a collapsed z stack image was generated in Fiji/ImageJ. For identification of neurons carrying transgene expression different marker lines were used, as well as the multicolor reference worm NeuroPAL^44^ (for full list of lines see key resources table).

#### Immunoprecipitation from *Xenopus* oocytes

IP experiments were performed on lysis from *Xenopus* oocytes expressing GFP tagged wild-type LGC-50, LGC-50 Δ363-379 or un-injected oocytes as an IP control. Lysis buffer (0.3 M sucrose, 10 mM sodium phosphate (pH 7.4)), was supplemented with Halt Protease and phosphatase inhibitor 1:100 (Thermo Fischer Scientific) and NP40 at an end concentration of 0.5% (Sigma Aldrich) immediately before usage. Oocytes were homogenized with 20 strokes in a glass homogenizer on day 3 after injection, keeping everything on ice. The lipid fraction was removed by centrifugation at 3000 x g at 4°C for 10 minutes. The lipid layer was removed and the remaining total lysate used for IP. 25 mL of equilibrated GFP-Trap MA beads (ChromoTek, GmbH) was incubated with 100 mL lysate at 4°C for 2h and then washed three times in TBS. Purified complexes were eluted from beads using Bolt LDS sample buffer and Bolt reducing agent (Thermo Fischer Scientific) at 70°C for 10 min before fractionated by SDS-PAGE (TGX Stain-free gel 4%–20%, Bio-Rad).

#### Immunoblotting

After SDS-PAGE electrophoresis the TGX stain-free gel was activated by UV in Gel Doc EZ (Bio-Rad) for quality control and the proteins transferred to a nitrocellulose membrane (Amersham Protran, 0.45 mm, ThermoFisher Scientific) with a wet transfer, (300 mA, 1 hour, Bio-rad). Membranes were blocked for 30 min in 5% milk and then incubated with primary antibody at 4°C overnight (anti-GFP HRP conjugated, A10260, diluted 1:1000 in TBS-T, ThermoFischer Scientific). The excessive unbound antibody was washed off the membrane using TBS-T before detection with Pierce ECL western blotting substrate (ThermoFisher Scientific). Blots were imaged using a ChemiDoc MP and Image Lab (Bio-Rad).

#### Aversive olfactory training and learning assays

The aversive olfactory training with the pathogenic bacteria strain *P. aeruginosa* PA14 and the analysis of learning were performed similarly as previously described^3^^,^^4^^,^^11^^,^^12^. Adult *C. elegans* hermaphrodites cultivated under standard conditions were transferred onto a training plate, which was prepared by inoculating a NGM plate with fresh overnight culture of PA14 in NGM medium and incubating at 26°C for two days, or onto a control plate, which was prepared by inoculating a NGM plate with fresh overnight culture of OP50 in NGM followed by two-day incubation at 26°C. The worms were trained for 4-6 hours at room temperature before learning assay. This exposure time has been shown previously to evoke robust conditioning without significantly impairing viability^3^^,^^11^.

To measure olfactory steering, a drop of 10 μL supernatant of a fresh overnight culture of PA14 or OP50 was put in the center of a 10 cm NGM plate. One naive or trained worm was placed 1.5 cm away from the supernatant before the recording started. The chemotactic steering of the worm was recorded by a Grasshopper3-GS3-U3-120S6M-C camera (FLIR Integrated Imaging Solutions) at 7 frames per second and analyzed by Wormlab (MBF Biosciences) and a MATLAB code^12^. Each worm was recorded until it reached the bacterial culture supernatant. If a worm did not reach the supernatant after 5 minutes, the recording stopped. The navigation index and the traveling distance (mm) between the start position and the endpoint were calculated^12^. Occasionally, the endpoint was outside of the supernatant drop. To measure the olfactory preference between PA14 and OP50, a droplet preference assay was performed similarly as described^11^. NGM cultures of OP50 and PA14 (overnight at 26 °C) were used to produce olfactory stimuli. Two alternating air streams odorized with OP50 or PA14 by passing through the bacterial culture were delivered to worms each swimming in a droplet of 2 μL of NGM buffer. The air streams alternated every 30 s to deliver the odorants of OP50 or the odorants of PA14 to the tested worms. The behavior of the tested worms was recorded and large body bends were counted^11^. The choice index and learning index were defined as: Choice index = (turning rate to OP50 − turning rate to PA14)/(turning rate to OP50 + turning rate to PA14); Learning index = Choice index of naive animals − Choice index of trained animals. To measure the olfactory preference between PA14 and OP50 using a two-choice assay on a plate^4^^,^^12^, 20 μL of fresh overnight culture of PA14 and 20 μL of fresh overnight culture of OP50 were placed on the opposite sides of a standard chemotaxis plate^54^ and dried on bench to form thin lawns before use. The concentration of the cultures was adjusted to optical density 600 = 1. Naive or trained worms were washed off respectively from the control or training plate and placed in the center of the testing plate, equidistance from the bacterial cultures, to test their olfactory preference between the two bacteria lawns. The number of the worms on each bacteria lawn was counted by the end of the assay to calculate learning index^4^.

#### Thermotaxis

The behavior was briefly adapted from Luo et al.^27^ and Mori and Ohshima^28^. In short, staged worms (L4 stage) were placed overnight at three different temperatures (15°C, 20°C and 25°C). The following day worms were tested on a specially built thermogradient equipment, that held a gradient from 15°C to 25°C vertically across the plate. The thermo stage had the size of a standard 96-well plate (128 × 85,5 mm), and rectangular four-well dishes (Nunc, ThermoFisher Scientific) filled with NGM agar were used for the testing. The worms were carefully washed in M9 before placed on testing plates to remove all bacteria. Worms were allowed to move freely over the temperature gradient for 1h after which the positions of the worms were scored.

### Quantification and statistical analysis

All values are shown as mean ± standard error of the mean (SEM).

#### Phylogenetic analysis of *C. elegans* LGC genes

A set of 171 LGC protein sequences were submitted to MAFFT multiple alignment server^55^ using the L-INS-i method for sensitive alignment. The resulting alignment in CLUSTAL format was refined with trimal^48^ using the parameters -gt 0.5 -w 7 to select only those alignment columns where considering the average of ± 7 positions, 50% of sequences were devoid of gaps. The trimmed alignment file was converted to PHYLIP format using an online server (http://sequenceconversion.bugaco.com/converter/biology/sequences/). The alignment in PHYLIP format was submitted to the PHYML-SMS web server^56^ which predicted the LG +G+F as the optimal model for building a phylogenetic tree. Finally, RAxML v.8 was used to build a tree^47^ using the PHYLIP format trimmed alignment with the following parameters -f a -m PROTGAMMAILGF -p 12345 -x 12345 -# 1000, which runs the program using fast bootstrapping with the LG +G+F model at 1000 bootstraps. Phylogenetic tree visualization was built in FigTree^45^, collapsing multiple isoforms of the same gene together, and colored by subgroup.

#### TEVC data analysis and plotting

Peak current was calculated using different software depending on origin of the data, manual recordings were analyzed with WinWCP and Robocyte2 collected data was analyzed with Stimfit or Robocyte2+. In all cases the peak current was taken during the window of interest.

Dose response and antagonist response curves were generated using custom-built python scripts (key resources table), which combined data from multiple recordings and normalized data by calculating I/Imax for each oocyte. Normalized mean, SD and n numbers where then imported into GraphPad where data was plotted and EC_50_ or IC_50_ values were calculated by fitting to the Hill equation using either three or four parameter slopes to obtain the highest degree of fit.

Ion selectivity analysis was performed using a custom-built python script (key resources table). Data was first normalized by calculating I/Imax for each oocyte and subtracting baseline currents from agonist induced currents in each solution. Non-linear quadratic line fitting was performed and reversal potential (E_Rev_) was calculated from the x intercept for each oocyte in each solution. Reversal potential shift (ΔE_Rev_) between ND96 and NMDG (Na^+^ free) and ND96 and Na Gluconate (low Cl^-^) solution was calculated for each oocyte and the individual values or mean, SD and n for each construct imported in GraphPad for plotting and statistical analysis. Statistically significant differences in ΔE_Rev_ were calculated in GraphPad using a 2-way ANOVA with Tukey’s correction for multiple comparisons. A selected representative trace, normalized by I/Imax and baseline subtracted, for each construct was also exported from python into GraphPad for plotting.

Repeated stimuli protocols were analyzed by calculating peak current for each agonist window in Stimfit and exporting data to GraphPad, where data was plotted and significance was tested using 2way ANOVA with Tukey’s multiple comparison correction.

#### Image analysis

Confocal images of worms exposed to *E. coli* OP50 or the pathogenic bacteria *Pseudomonas aeruginosa* PA14 for 6h were analyzed by calculating an intensity ratio between nerve ring and a posterior region next to the nerve ring using Fiji/ImageJ. Images were collapsed into one combined z stack prior analysis. Intensity histograms across the entire worm was also analyzed using the histogram tool in Fiji/ImageJ.

#### Behavioral Analysis

The olfactory steering of the worm was recorded by a Grasshopper3-GS3-U3-120S6M-C camera (FLIR Integrated Imaging Solutions) at 7 frames per second and analyzed by Wormlab (MBF Biosciences) and a MATLAB code^12^. For two-choice assays, the number of the worms on each bacteria lawn was counted by the end of the assay to calculate learning index^4^.

## Data Availability

All experimental data, including results of electrophysiological, behavioral, imaging, and biochemical experiments, will be shared by the lead contact upon request. All original code has been deposited at GitHub and Zenodo and is publicly available as of the date of publication. DOIs are listed in the key resources table. Any additional information required to reanalyze the data reported in this paper is available from the lead contact upon request.

## Supplemental information

Document S1. Figures S1–S6

Table S1. List of *C. elegans* genes used for phylogenetic analysis, related to Figure 1

Table S2. Dataset generated using NetPhos 3.1 mapping potential phosphorylation sites in LGC-50, related to Figure 5

Table S3. Primers used for genotyping, related to STAR Methods

Document S2. Article plus supplemental information
