# Supplementary material for: Deorphanization of novel biogenic amine-gated ion channels identifies a new serotonin receptor for learning
Source: Curr Biol. 2021 Oct 11;31(19):4282–4292.e6. doi: 10.1016/j.cub.2021.07.036 (PMC8536830; doi:10.1016/j.cub.2021.07.036)
Supplement: Document S1. Figures S1–S6 [file mmc1.pdf]

Current Biology, Volume *31*

Supplemental Information

Deorphanization of novel biogenic amine-gated ion channels identifies a new serotonin receptor for learning

Julia Morud, Iris Hardege, He Liu, Taihong Wu, Myung-Kyu Choi, Swaraj Basu, Yun Zhang, and William R. Schafer

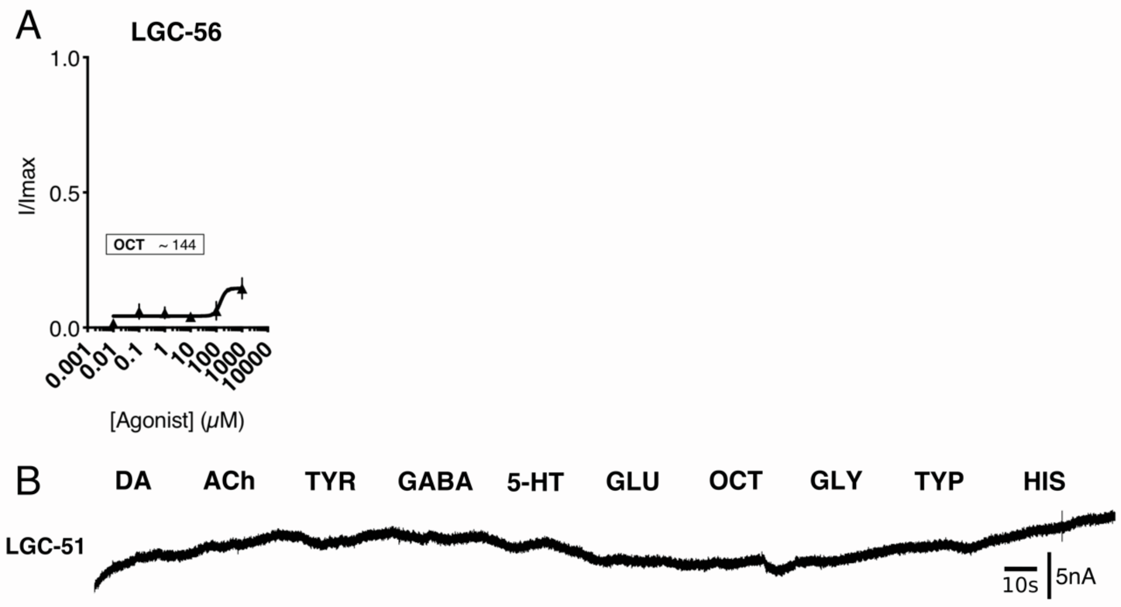

Figure S1: Octopamine response in LGC-56 and non-responsiveness of a LGC-51-expressing oocyte, related to Figure 2 (A) Dose response for octopamine application on LCG-56 (previously named GGR-3). (B) Agonist panel application on an oocyted expressing LGC-51 homomer shows no responses to the possible agonists evaluated.

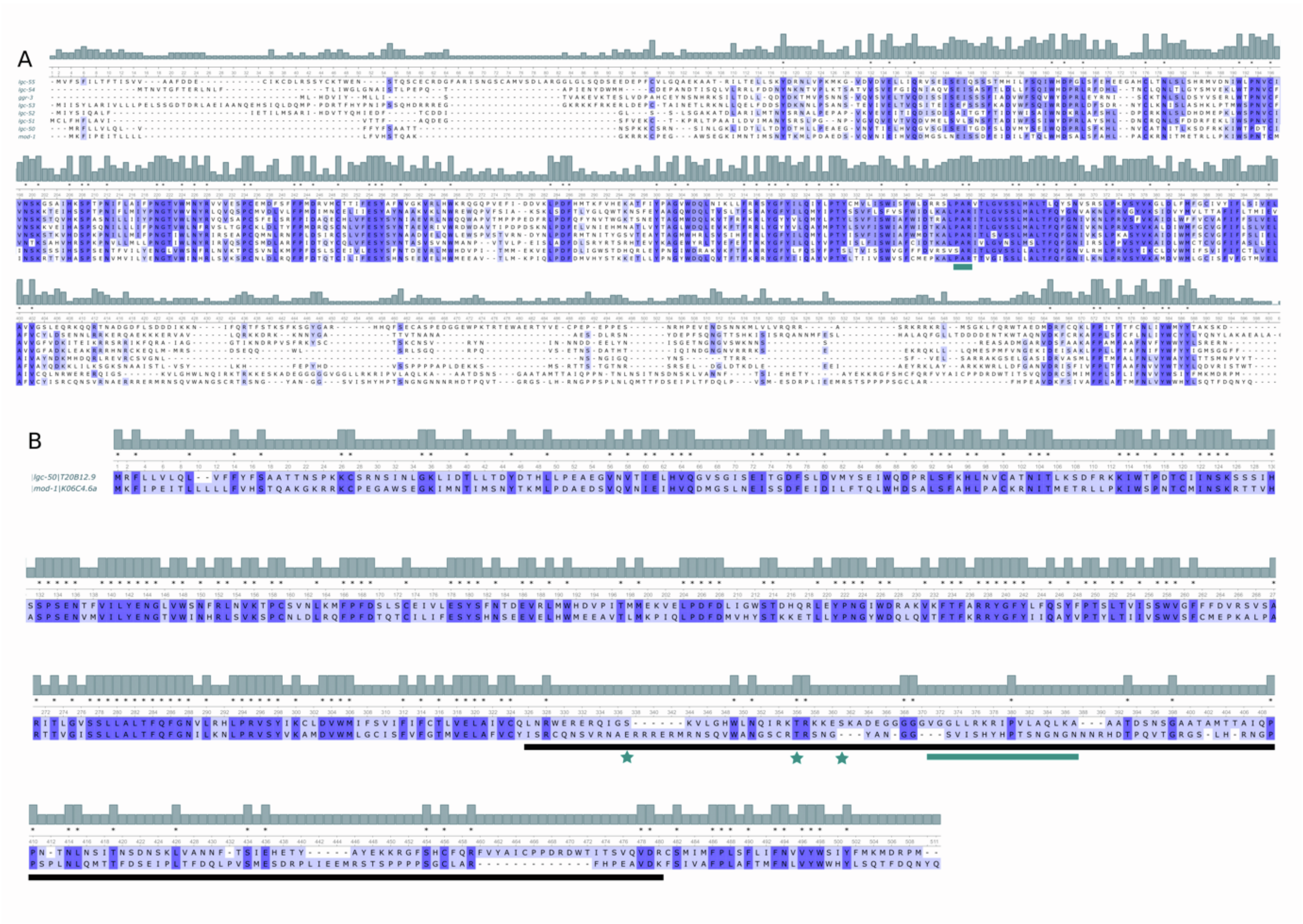

Figure S2: Alignment of LGIC polypeptide sequences from *C. elegans*, related to Figure 2 (A) Alignment of aminergic LGCs shows at the green line conservation of the PAR motif (position 348) for all channels but LGC-50, which has a point mutation replacing proline with serine. (B) Alignment of LGC-50 and MOD-1 shows high homology until the start of the M3/4 loop, which is indicated by the black line. The green stars indicate the predicted phosphorylation sites in LGC-50 (Table S2) and the green line marks the deleted region in LGC-50(Δ363-379), that when deleted allows LGC-50 to traffic to the plasma membrane.

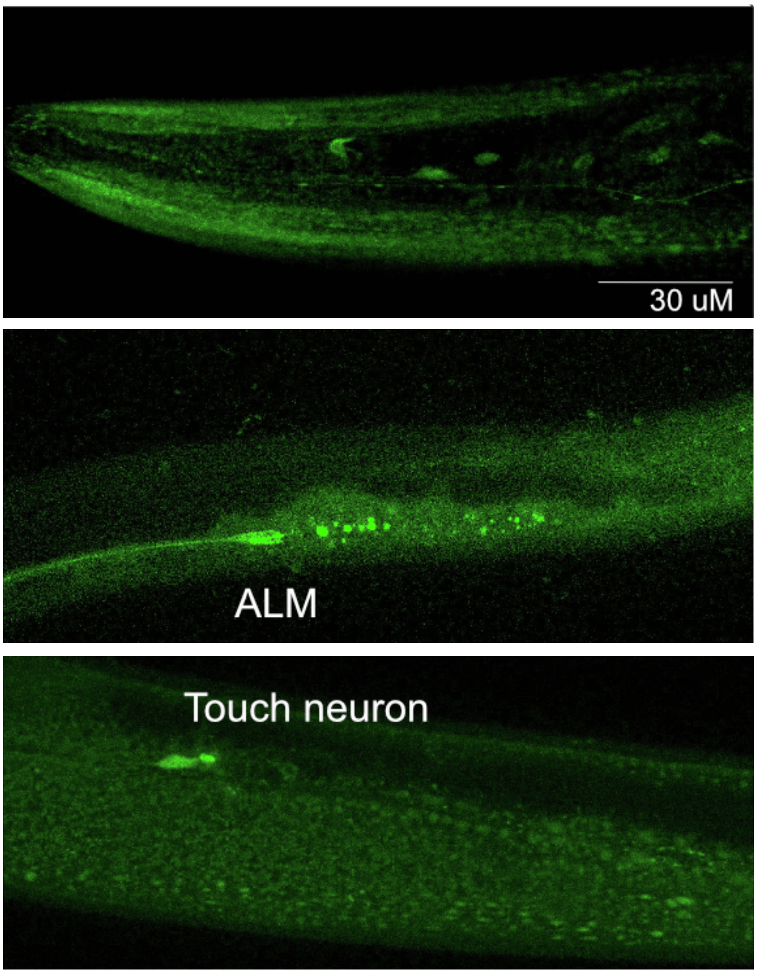

Figure S3: Identification of *lgc-56*-expressing cells using a CRISPR-inserted reporter, related to Figure 3 (A)Confocal images of *lgc-56::SL2 mNeonGreen(syb2794)* CRISPR insertion strain indicating expression in touch neurons and as well as several yet unidentified head neurons. In all images, left is anterior, up is (roughly) dorsal. Images indicate the head (top), anterior midbody (middle) and posterior midbody (bottom) of a single animal.

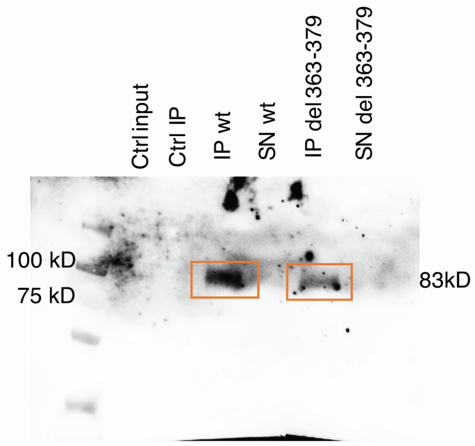

Figure S4: Expression of LGC-50::GFP and LGC-50Δ363-379::GFP in oocytes, related to Figure 5 Shown is a Western blot image following immunoprecipitation from oocytes expressing either LGC-50::GFP or LGC-50Δ363-379::GFP using anti-GFP antibody detection. Contro input is total lysate from untreated oocytes. IP: immunoprecipitation using anti-GFP magnetic beads, SN: supernatant after IP.

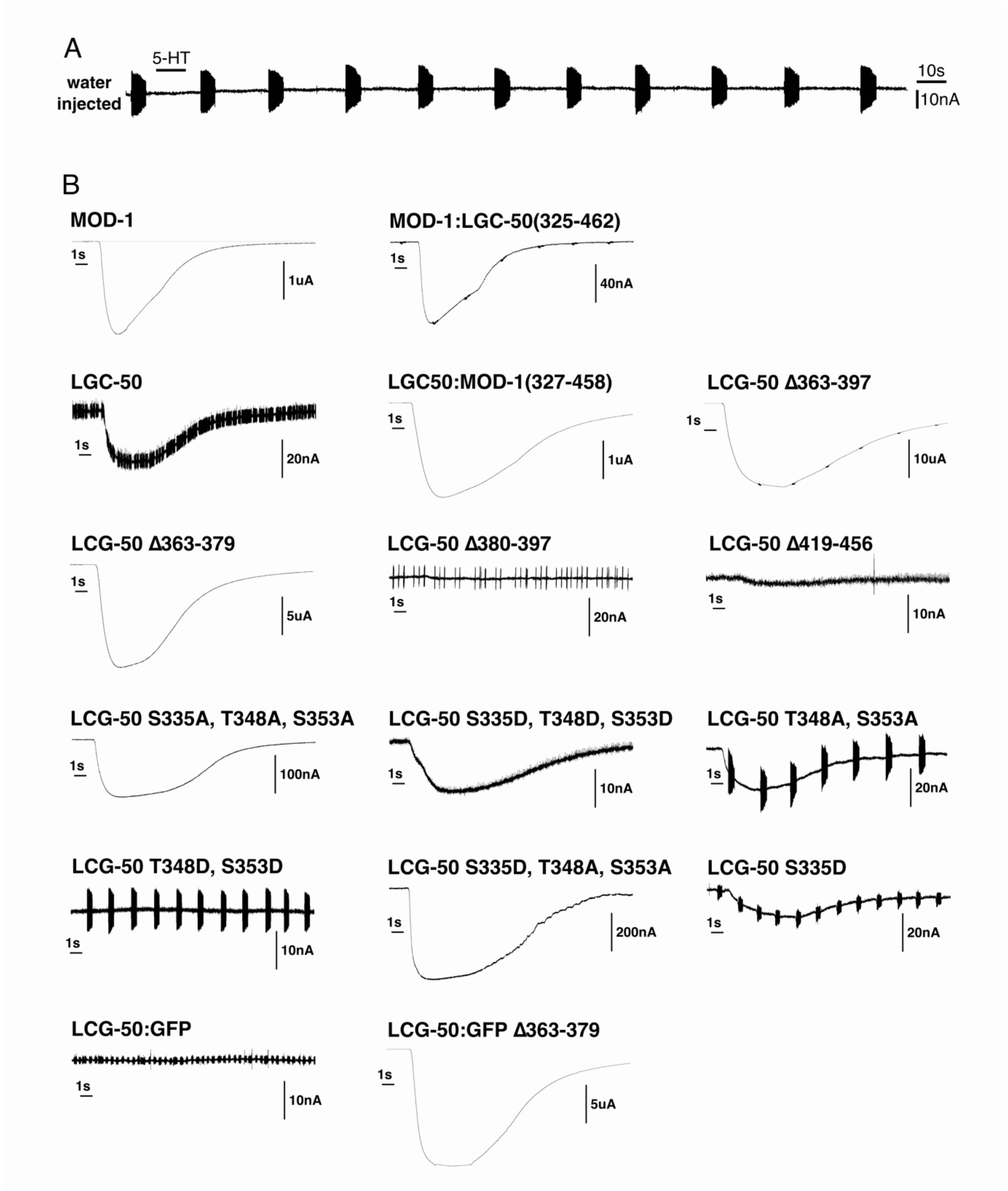

Figure S5: Representative traces from two-electrode voltage clamp recordings of water-injected oocytes and oocytes injected with LGC-50 chimeric proteins, related to Figure 5 (A) TEVC trace from water injected oocytes shows no response to 5-HT application. (B) Representative traces from TEVC recordings in oocytes from different chimera proteins between MOD-1 and LGC-50, as well as several LGC-50 deletion and phosphomimic mutants.

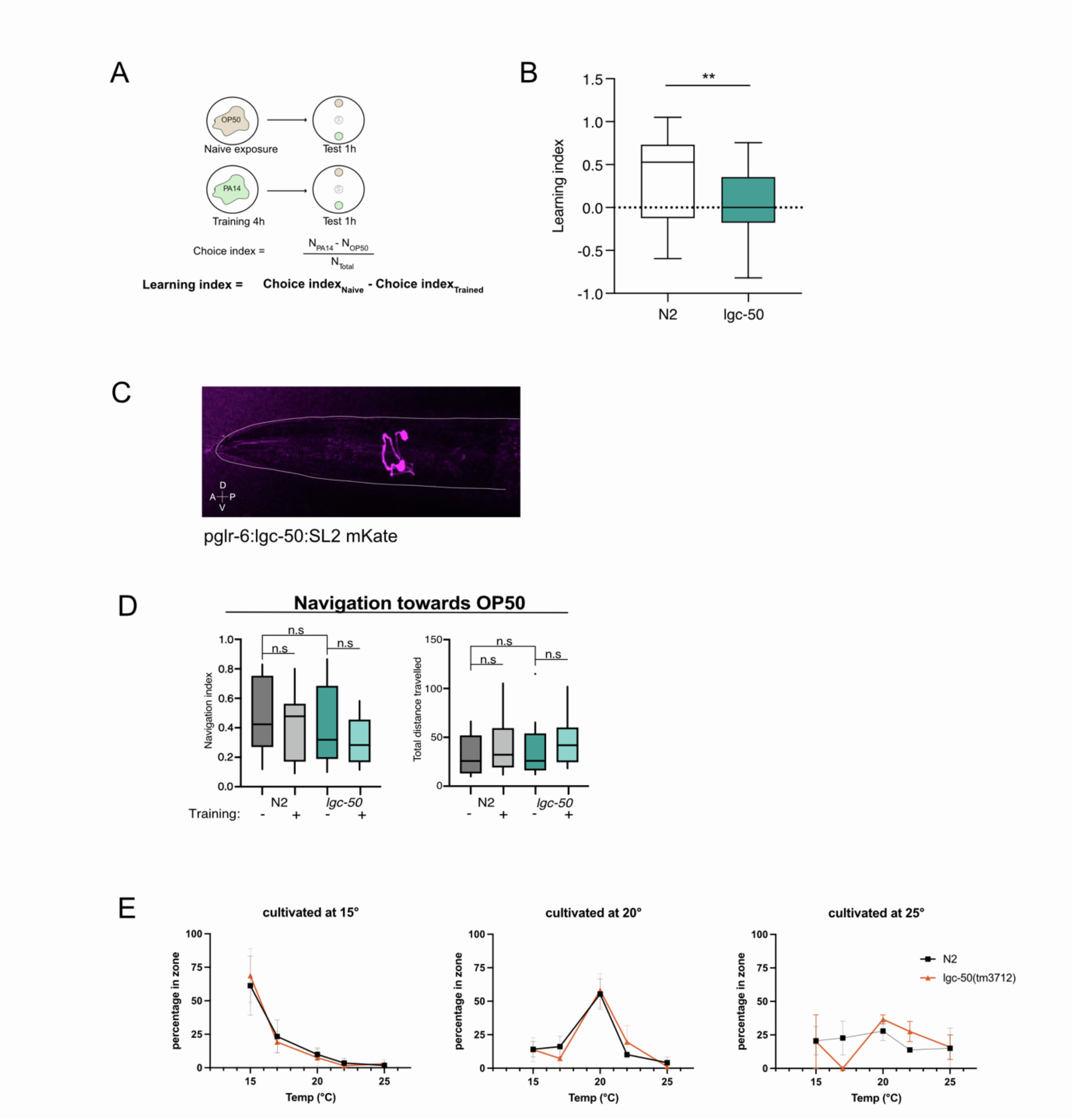

Figure S6: Additional behavioural characterization of *lgc-50* mutants, related to Figure 7 (A) Schematic description of two-choice aversive learning assay. (B) *lgc-50(tm3712)* is defective in the two-choice learning assay and significantly different from wild-type animals. (n = 31 plates tested for N2 and 41 plates tested for *tm3712*, 50-200 worms per plate). Student's t test, ** $p < 0.01$. Boxplots show first and third quartile, median, and the whiskers extend to data points that are equal or less than 1.5 IQR from the quartiles. Dots represent outliers. (C) Confocal image showing that expression of *lgc-50* under the *glr-6* promotor is cell-specifically localised in RIA. (D) *lgc-50* mutants display normal chemotaxis towards the non-pathogenic *E. coli* OP50. (n = 17 naive N2 and 16 trained N2; n = 17 naive *lgc-50* and 15 trained *lgc-50*). Two-way ANOVA with Tukey's multiple comparisons test, n.s., not significant; boxes show first and third quartile, median and the whiskers extend to data points that are equal or less than 1.5 IQR from the quartiles. Dots represent outliers. (E) Thermotaxis data comparing N2 and *lgc-50* mutant worms after cultivating the worms at three different temperatures overnight and performing 1h of thermotaxis testing the following day. No difference in thermotaxis behaviour was detected. (n for cultivation at 15°C: N2=46, *tm3712*=51, 20°C: N2=37, *tm3712*=63, 25°C: N2=68, *tm3712*=35).
